# Optimizing adolescent HIV care: a review of EMR system quality for clinical monitoring in Zambia

**DOI:** 10.3389/fpubh.2026.1716382

**Published:** 2026-03-06

**Authors:** Kaala Moomba, Brian Van Wyk

**Affiliations:** School of Public Health, Faculty of Community and Health Sciences, University of the Western Cape, Cape Town, South Africa

**Keywords:** adolescents, clinical, data quality, electronic medical records, HIV, retention, viral suppression, Zambia

## Abstract

Adolescents living with HIV (ALHIV) in Zambia experience poorer treatment outcomes than adults, with lower viral suppression and higher loss to follow-up rates. Electronic medical record (EMR) systems such as SmartCare aim to strengthen patient monitoring, but their utility is contingent on high data quality. Accurate monitoring of ALHIV treatment outcomes is critical for improving patient care and supporting progress toward UNAIDS 95–95-95 targets. We conducted a retrospective cross-sectional review of EMR data for ALHIV on antiretroviral therapy in selected Lusaka facilities (January–December 2023). Data were extracted from SmartCare and assessed using the WHO Routine Data Quality Assessment framework across three dimensions: completeness, correctness, and consistency. Records from 3,978 ALHIV were analysed. Socio-demographic variables (gender, date of birth, age at ART initiation) and treatment data (ARV regimen) performed strongly, with ≥98% completeness, correctness, and consistency. In contrast, clinical variables showed substantial gaps. Completeness for baseline (*n* = 1,707) and current (*n* = 2,149) CD4 counts was 43% and 54%, respectively, though correctness and consistency exceeded 99%. Pregnancy and breastfeeding data among female adolescents (*n* = 2,177) were particularly poor, with completeness of 4% and 12%. By comparison, history of tuberculosis (100%) and current viral load results (91%) were reliably captured. Whereas SmartCare demonstrated strong reliability for demographic and treatment indicators, notable weaknesses in the completeness of key clinical variables, such as CD4 count and pregnancy/breastfeeding status were observed. These gaps may reflect variability in data entry workflows and system-level factors, including EMR upgrades, highlighting areas for targeted improvement. We recommend targeted training, system redesign to enforce mandatory entry of critical fields, and routine data quality monitoring to ensure EMR systems provide accurate and actionable data. Addressing these gaps would facilitate optimising HIV care and support progress toward achieving the UNAIDS 95–95-95 goals for ALHIV in Zambia.

## 1 Introduction

HIV/AIDS remains a major public health concern in Zambia, with an annual incidence of 0.31% among adults aged 15 years and older, corresponding to approximately 28,000 new infections each year. Women are disproportionately affected, with an incidence of 0.56% compared to 0.06% among men. Similarly, overall HIV prevalence in this age group is 11.0%, with higher rates among women (13.9%) than men (8.0%) (1). Within this broader epidemic, adolescents and young people (AYP) represent a critical population in the HIV epidemic, as they are at heightened risk of new infections and face unique barriers to care (2). According to the 2018 Zambia Demographic and Health Survey, HIV prevalence among individuals aged 15–24 years was 3.8%. Among those aged 15–19 years, prevalence was 1.9%, with higher rates among women (2.6%) compared to men (1.2%) (3). Despite Zambia’s commitments to global HIV targets such as the UNAIDS 95–95-95 goals, which aim to end the epidemic by ensuring that 95% of people living with HIV know their status, 95% of those diagnosed are on treatment, and 95% of those on treatment achieve viral suppression, adolescents continue to lag behind. They experience higher rates of loss to follow-up, suboptimal viral suppression, and poorer health outcomes compared to adults (4, 5). For example, regional programmatic data indicate that viral suppression among adolescents aged 10–19 years in sub-Saharan Africa ranges between approximately 65–75%, compared to over 85–90% among adults, while loss-to-follow-up rates among adolescents may be 1.5–2 times higher than those observed in adult populations (6–10).

Addressing these gaps requires not only tailored models of care but also robust health information systems that can reliably monitor treatment patterns, identify adolescents at risk of poor outcomes, and generate actionable insights. In this context, electronic medical record (EMR) systems are a critical tool for optimizing adolescent HIV care by strengthening clinical monitoring and enabling data-driven interventions.

The introduction of EMR in developing countries is widely recognized as a strategy to strengthen health systems by improving data quality and enhancing the delivery of healthcare services. EMRs provide complete, accurate and timely information, offering the potential to improve the quality of patient care, reduce the workload of healthcare workers, facilitate monitoring and evaluation of health programs, and generate real-time data for evidence-based decision-making (11–14). As a result, governments in these regions are making significant investments in EMR systems to strengthen healthcare delivery and boost the overall efficiency of public health facilities (15). With this growing availability of large EHR databases, clinical researchers are increasingly interested in the secondary use of clinical data (16). The government of Zambia has invested heavily in EMR systems to strengthen healthcare delivery and improve viral load monitoring 2005 (17). The Zambian Ministry of Health’s Digital Health Strategy 2022–2026 emphasizes the integration and strengthening of electronic health systems to improve data management, patient monitoring, and healthcare service delivery (18). This strategy aligns with the adoption of EMR for clinical monitoring, aiming to enhance data accuracy, streamline patient care, and support evidence-based decision-making. Zambia has made strides in improving the management of patients through adoption of an EMR, called SmartCare. Over time, the EMR has evolved from its initial version, SmartCare Legacy, to SmartCare Plus, and in the last 8 months to the advanced SmartCare Pro, with enhancements designed to adapt to current healthcare needs (17).

Accurate, reliable, and timely data are essential to realizing the full benefits of EMRs. High-quality data are characterized by accuracy, reliability, relevance, and fitness for use (19–21), all of which are critical for effective service delivery, informed decision-making, and program monitoring necessary to maintain high healthcare standards. Yet, despite this importance, many settings, including Zambia, continue to report challenges with EMR data quality (22–24). Contributing factors include insufficient training, staffing shortages, and broader health system challenges such as unstable electricity supply (20). Addressing these gaps requires regular monitoring and continuous improvement of EMR data quality to ensure reliable evidence for decision-making and program management.

Optimizing care for adolescents living with HIV (ALHIV) requires health information systems that can generate reliable, timely, and actionable data for clinical monitoring and programmatic decision-making. While Zambia has invested heavily in electronic medical record (EMR) systems, such as SmartCare, to strengthen patient management and improve viral load monitoring, persistent challenges with data quality, including inaccuracy, incompleteness, and inconsistency undermine the potential of these systems to support adolescent HIV care. Without addressing the quality of EMR data, the promise of these systems to improve clinical monitoring, inform tailored interventions, and ultimately optimize adolescent HIV care will remain unrealized.

This paper reports on the quality of the EMR system for clinical monitoring among ALHIV in Zambia’s public health sector. The assessment evaluated key data quality dimensions which included accuracy, completeness, correctness, and consistency in order to identify gaps and generate recommendations for strengthening EMR systems in order to enhance patient care and overall health system performance.

## 2 Materials and methods

### 2.1 Study design

A retrospective cross-sectional study design was used to ascertain accuracy, completeness, correctness, and consistency of the EMR data for ALHIV on ART.

### 2.2 Study population

The study population included ALHIV receiving ART from selected health facilities in Lusaka district from January 2023–December 2023 and meeting the eligibility criteria.

### 2.3 Selection of health facilities

Health facilities were purposively selected to support a pragmatic assessment of the performance and data quality of the EMR system used for routine clinical monitoring of adolescents living with HIV (ALHIV) in Zambia’s public health sector. Facilities were eligible for inclusion if they: (i) implemented the national EMR platform for HIV care and treatment, (ii) provided ART services to adolescents, (iii) routinely captured viral load and clinical monitoring data within the EMR and (iv) had sufficient duration of EMR use to allow assessment of data quality over time.

Selection aimed to capture variation in implementation context, including differences in facility level and patient volume to assess EMR data quality under routine service delivery conditions. High-volume ART facilities were prioritized to ensure the availability of an adequate number of adolescent records for evaluating key data quality dimensions including accuracy, completeness, correctness and consistency across the clinical monitoring cascade.

### 2.4 Sample size and representativeness

A formal statistical sample size calculation was not undertaken, as the primary objective of the study was to evaluate health information system performance and data quality, rather than to estimate population-level clinical outcomes. The analytical sample was therefore defined by the number of eligible ALHIV records available in the EMR within the selected facilities during the study period. This approach is consistent with health technology implementation and digital health evaluation studies, where the unit of inference is the functionality and reliability of the digital system rather than individual-level effect estimation. Although the findings are not intended to be statistically representative of all health facilities in Zambia, inclusion of multiple public-sector ART sites using the national EMR platform enhances analytical generalizability. The results are transferable to similar implementation settings where EMRs are used for routine HIV clinical monitoring and they provide actionable insights for strengthening EMR system performance and data use within comparable low-resource health system contexts.

### 2.5 Data collection and management

#### 2.5.1 Data sources and extraction

Routine data were extracted from SmartCare, a widely used electronic health record (EHR) system in Zambian public health facilities. Implementation varies by site: some use an “electronic last” (e-last) model, where designated personnel enter data retrospectively, while others adopt an “electronic first” (e-first) approach, with healthcare providers inputting data in real time at service points. In August 2024, anonymized data were extracted using unique patient identifiers, and consisted of demographic, clinical and treatment information from routine ART visits.

**Figure 1:**
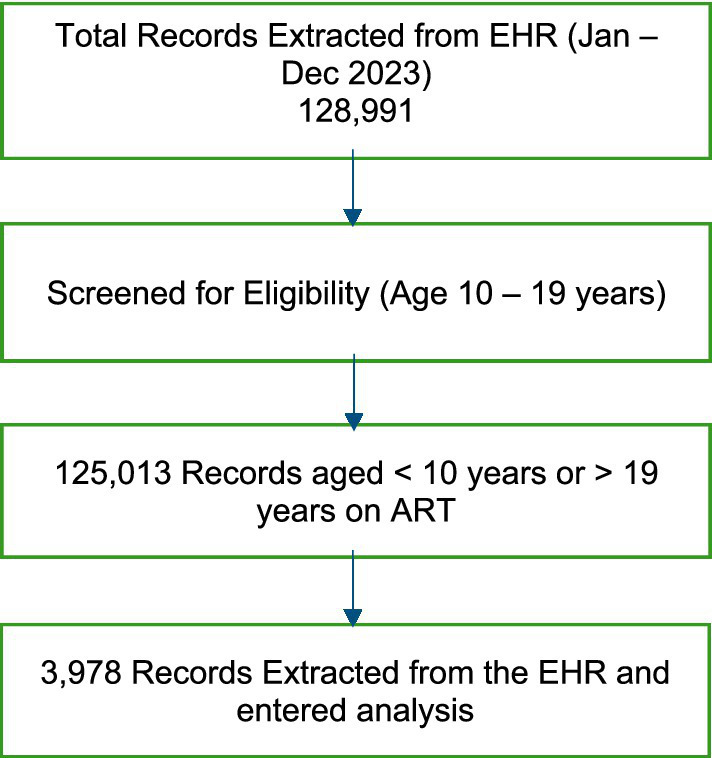
Selection process of ALHIV records.

Figure 1 illustrates the selection process of ALHIV records included in the analysis. Of all adolescent records available in SmartCare during January–December 2023, 3,978 met the eligibility criteria and were included in the final analysis. Records were included if they (i) were aged 10–19 years during the study period, (ii) were documented as receiving ART and (iii) had a valid unique patient identifier. Records not meeting age criteria or lacking ART documentation were excluded prior to analysis.

As all ART data are recorded in SmartCare, any missing data could not be retrieved. Sociodemographic variables included gender, date of birth and age at ART initiation. Clinical characteristics covered baseline WHO stage, baseline and current CD4 count, pregnancy, breastfeeding, history of active tuberculosis (TB) and current VL result. Treatment characteristics included current ARV regimen.

The extracted data were entered into a Microsoft Excel (Microsoft Corporation, Washington, DC, United States) spreadsheet, cleaned, and saved into a password-protected excel file to prevent any unauthorized access or alterations of the data. The excel spreadsheet was imported into the SPSS statistical software (IBM SPSS version 29.0.2.0, IBM Corp. USA) for analysis.

#### 2.5.2 Operational definitions of data quality dimensions

Data quality was assessed using three core dimensions; completeness, correctness and consistency predefined and applied uniformly across socio-demographic, clinical and treatment variables extracted from the EMR system (Table 1).

**Table 1:** Assessment guide.

Characteristic	Variable	Data completeness	Data correctness	Data consistency
Socio-demographic	Gender	Check if gender is recorded for all ALHIV	Check if all values entered match the allowable categories (e.g., Male, Female)	Check if gender is uniform across different system modules
	Date of Birth	Check if date of birth is recorded for all ALHIV	Check if DOB values are logical (e.g., not in the future, not unrealistically old/young for ALHIV)	Check if DOB remains the same across visits and modules (no changes over time unless correcting an error)
	Age at ART Initiation	Check if age at ART initiation is recorded for all ALHIV	Check if all records are within realistic bounds (not negative, not older than current age)	Check if age at ART initiation is consistent with DOB and ART start date
Clinical	Baseline WHO Clinical Stage	Check if WHO clinical stage is recorded for all ALHIV	Check if WHO stage is formatted consistently and falls within 1–4	Check if baseline WHO stage remains constant over time (should remain the same).
	Baseline CD4 Count	Check if baseline CD4 count is recorded for all ALHIV	Values must be biologically plausible (e.g., 0–3,000 cells/μL; avoid negative).	Check if baseline CD4 count remains constant over time (should remain the same).
	Current CD4 Count	Check if all ALHIV have a most recent CD4 count recorded in the EMR	Values must be biologically plausible (e.g., 0–3,000 cells/μL; avoid negative)	Check if most recent CD4 count is chronologically later than baseline and align with visit/lab test dates.
	Pregnancy	Check that pregnancy status is recorded for all female ALHIV	Check that pregnancy is only applicable for female ALHIV	Ensure pregnancy status aligns with other clinical data (e.g., ARV regimen)-according to national guidelines
	Breastfeeding	Check that breastfeeding status is recorded for all female ALHIV	Check that breastfeeding is only applicable for female ALHIV	Ensure breastfeeding status aligns with other clinical data (e.g., ARV regimen)-according to national guidelines
	History of Active TB	Check if TB history is recorded for all ALHIV	Check that the ‘History of Active TB’ variable accurately reflects documented clinical TB diagnosis	Check if once TB history is documented as yes, it remains yes unless it was an error and is corrected
Treatment	ARV Regimen	Check if ARV regimen is recorded for all ALHIV	Check if regimen consists of valid, nationally approved combinations (e.g., TDF/3TC/DTG, ABC/3TC/EFV). No invalid entries (e.g., single drug alone)	Check if Initial regimen remains the same unless a documented switch is recorded and the regimen history should align with age according to national guidelines

Data completeness was defined as the presence of a required data element within the EMR for an eligible ALHIV. A variable was considered complete if a valid entry was recorded (not missing or blank) for each ALHIV record assessed. Completeness was evaluated at the individual-variable level and summarized as the proportion of records with non-missing values.

Data correctness was defined as the validity and plausibility of recorded values relative to allowable categories, biological plausibility, temporal logic and national HIV treatment guidelines. Correctness checks included verification that categorical variables (e.g., sex, WHO clinical stage, ARV regimen) conformed to predefined allowable values; numeric variables (e.g., age, CD4 counts) fell within biologically plausible ranges; and clinical variables (e.g., pregnancy and breastfeeding status) were logically applicable to the patient’s sex and clinical context.

Data consistency was defined as the internal agreement of the same data element across time, visits and EMR modules, as well as logical alignment between related variables. Consistency checks assessed whether static variables (e.g., sex, date of birth, baseline WHO stage, baseline CD4 count) remained unchanged over time unless corrected, whether temporally linked variables were chronologically coherent (e.g., age at ART initiation consistent with date of birth and ART start date) and whether clinical and treatment variables aligned with national guidelines (e.g., pregnancy status and ARV regimen appropriateness).

These operational definitions guided systematic review of EMR records and ensured standardized assessment of data quality across all included facilities and variables.

### 2.6 Error and inconsistency detection procedures

Data quality assessment followed a rule-based, reproducible validation workflow adapted from the WHO Data Quality Review (DQR) Toolkit and Zambia national HIV treatment guidelines. All checks were predefined and applied uniformly across facilities using Microsoft Excel and IBM SPSS.

#### 2.6.1 Step 1: completeness checks

Mandatory variables were screened for missing, blank or null values. Records with absent required fields were flagged as incomplete.

#### 2.6.2 Step 2: correctness checks

Values were assessed against predefined allowable categories, biologically plausible ranges and clinical logic. Categorical variables were validated against permitted value sets, numeric variables were evaluated using range checks and sex-specific variables (e.g., pregnancy, breastfeeding) were assessed for clinical applicability.

#### 2.6.3 Step 3: consistency checks

Internal agreement was assessed through cross-variable and longitudinal validation. Static variables were examined for unexpected changes over time, while temporally linked variables were evaluated for chronological coherence. Clinical and treatment variables were assessed for alignment with national HIV treatment guidelines.

#### 2.6.4 Step 4: flagging and classification

Records violating one or more validation rules were systematically flagged as having data quality issues. Variable-specific criteria guiding these checks are summarized in Table 1.

#### 2.6.5 SmartCare architecture and workflow

SmartCare is Zambia’s national electronic medical record (EMR) system for HIV care, designed to support routine clinical monitoring and reporting across public-sector health facilities. The system can operate in two primary modes: “Electronic-First” (e-first), where healthcare providers enter data in real time at service points and “Electronic-Last” (e-last), where designated data clerks enter data retrospectively from paper-based registers. However, the migration to SmartCare Pro, facilities are required to operate under the electronic-first (e-first) workflow model.

The EHR captures demographic, clinical, laboratory and treatment information for each patient and stores data in a centralized database accessible for analysis and reporting. Implementation and data entry workflows vary by facility, influenced by staffing, patient volume and local infrastructure. System upgrades, such as the SmartCare Pro rollout have occurred periodically, which may affect data completeness and consistency during transition periods. This architecture and variability in workflow provide important context for interpreting our findings.

### 2.7 Data analysis

This study applied the Routine Data Quality Assessment (RDQA) dimension of the WHO Data Quality Review (DQR) Toolkit to evaluate internal data quality within the EMR system.

- Completeness was assessed by checking that all pertinent information related to ALHIV patients was present. This involved identifying missing data elements (25–28).
- Correctness was assessed by checking the consistency of the formatting of various data elements ensuring reliability (29, 30).
- Consistency was examined by assessing inconsistencies in data elements (24, 31, 32). This process will ensure that the information aligns seamlessly and conforms to predefined standards within the national guidelines.

Since most health facilities operate paperless systems for HIV services, external validation with paper records was not feasible. Rather, the assessment focused on internal consistency within the EMR and compliance with national guidelines to identify and address potential data quality gaps.

A structured assessment guide (Table 1) was developed to enable a systematic and transparent analysis of the extracted data, ensuring consistency in evaluating data quality.

## 3 Results

Overall, socio-demographic variables demonstrated excellent performance across all three data quality dimensions, while several clinical and treatment-related variables revealed notable gaps in completeness and correctness (Table 2).

**Table 2:** Completeness, correctness and consistency of EHR data entry for HIV-related variables among ALHIV, January–December 2023 (*N* = 3,978).

Category of variables	n	Completeness	Correctness	Consistency
Socio-demographic				
Gender	3,978	100	100	100
Date of Birth	3,978	100	100	100
Age at ART initiation	3,978	100	100	100
Clinical				
Baseline WHO staging	3,508	88	100	100
Baseline CD4 Count	1,707	43	99	100
Current CD4 Count	2,149	54	99	100
*Pregnancy	87	4	100	
*Breastfeeding	261	12	100	
History of active TB	3,978	100	100	100
Current VL result	3,621	91	100	100
Treatment				
ARV regimen	3,978	100	98	100

VL, viral load; TB, Tuberculosis. For pregnancy and breastfeeding variables, *N* = 2,177 (*female ALHIV only). All other variables use the full cohort denominator (*N* = 3,978) unless otherwise specified.

**Figure 2:**
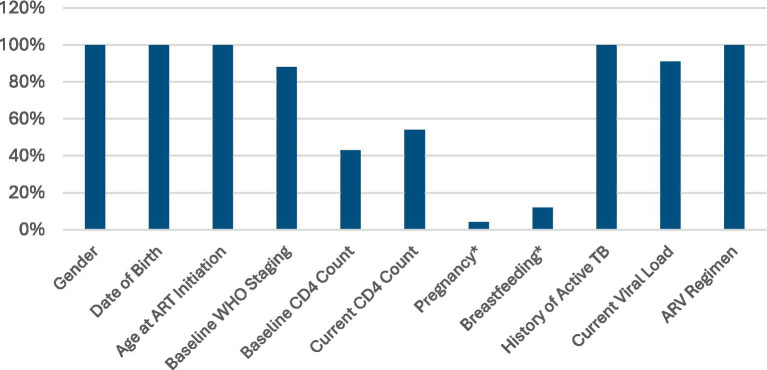
Comparative completeness of EMR variables (*Female ALHIV only, *N* = 2,177).

To enhance interpretability and strengthen the data-use agenda, Figure 2 presents a comparative visualization of completeness across key variables. The figure highlights the marked contrast between socio-demographic indicators (100% completeness) and critical clinical indicators, particularly pregnancy (4%) and baseline CD4 count (43%). This visual comparison underscores the magnitude of clinical data gaps affecting monitoring and programmatic decision-making for ALHIV.

### 3.1 Socio-demographic variables

Gender, date of birth, and age at ART initiation were fully captured across all 3,978 adolescents living with HIV (ALHIV), with 100% completeness, correctness, and consistency. This indicates strong system reliability for core demographic data.

### 3.2 Clinical variables

Completeness varied substantially across clinical indicators. Baseline WHO staging was recorded for 88% of participants, with complete correctness and consistency. In contrast, baseline and current CD4 counts had much lower completeness (43 and 54%, respectively), though both variables demonstrated very high correctness (≥99%) and complete consistency. Pregnancy and breastfeeding data, assessed only among females (*n* = 2,177), also showed notable gaps, with completeness at 4 and 12% and correctness at only 100% for both, respectively. History of active TB showed 100% completeness, correctness, and consistency. Current viral load results were available for 91% of ALHIV and, similar to other variables, demonstrated complete correctness and consistency.

### 3.3 Treatment variables

ARV regimen data were complete for all ALHIV (100%) and largely correct (98%), with excellent consistency (100%).

## 4 Discussion

In this study, we evaluated the quality of data in the EMR system for ALHIV using three key metrics: completeness, correctness, and consistency. Overall, socio-demographic and treatment-related variables fared very well, but several clinical indicators showed gaps, especially in completeness and correctness suggesting areas for targeted improvement.

Among socio-demographic variables, gender, date of birth, and age at ART initiation data quality was uniformly high: 100% completeness, correctness, and consistency. This mirrors findings from the Lusaka SmartCare EMR system, where registration data such as date of birth and identifiers were reported to be essentially complete and accurate under both “Electronic-Last” (paper to EMR) and “Electronic-First” (real-time entry) modes (33). Socio-demographic information such as age and date of birth is usually easier to capture and less subject to clinical complexity or changing status, which likely explains the high performance in this domain and in many EMR systems these socio-demographic fields are mandatory, further contributing to their completeness and correctness (34). In contrast, some clinical indicators showed weaker performance. Baseline WHO clinical stage was available for 88% of participants, with fully accurate and consistent entries. However, baseline and current CD4 counts had much lower completeness (43 and 54%, respectively), even though when recorded they tended to be correct and consistent.

The migration from SmartCare Legacy/Plus to SmartCare Pro may have contributed to observed gaps in data completeness. During EMR transitions, risks include unmapped legacy fields, non-migrated historical laboratory values, and temporary disruptions in data entry practices. SmartCare Pro introduced workflow standardization favouring the electronic-first model; however, certain clinical variables such as pregnancy and breastfeeding were not consistently configured as mandatory fields at the time of migration. In addition, where historical CD4 values were stored in legacy modules, incomplete field mapping during migration may have resulted in apparent missingness in the upgraded system.

These findings highlight an important lesson for countries undergoing EMR transitions: system upgrades must include rigorous data migration validation, field mapping audits, and reconfiguration of mandatory clinical indicators to prevent unintended data loss or under-capture during transition phases (35–37).

In other settings, EMR systems have struggled similarly with CD4 count data. For instance, in Kenya, an intervention of implementing cloud-based EMR reduced the proportion of missing baseline CD4 values from about 18 to 8%, but even after improvements, missingness remained a challenge (38). The problem is not unique to CD4; certain clinical and laboratory variables often suffer incomplete recording in Low and Middle Income Countries (LMIC) EMRs (39). Similarly, pregnancy and breastfeeding status among female ALHIV were particularly problematic with completeness for pregnancy and breastfeeding extremely low (4 and 12% respectively). These findings suggest that clinical complexity and EMR system constraints disproportionately affect completeness for dynamic or episodic clinical indicators.

The observed differences between e-first and e-last facilities reinforce the operational importance of workflow design. Real-time data entry reduces transcription loss, minimizes delayed documentation, and improves capture of episodic clinical events such as pregnancy and laboratory results. This provides actionable insight for facility managers and policymakers considering full transition to electronic-first models (33, 36, 40, 41).

History of active TB was well captured (100% across all three metrics), and current viral load results were available for 91% of adolescents, both with correct and consistent entries. Treatment variables, in this case ARV regimen data were also nearly complete and mostly correct (98%), with full consistency. These results follow trends seen in other studies like the Rwanda EMR assessment, where medication pick-ups and core treatment regimen fields had relatively strong completeness compared to more complex or episodic clinical measures (33, 36).

These gaps have implications for the UNAIDS 95–95-95 cascade. While high completeness of socio-demographic and treatment data supports monitoring the first and second “95” (diagnosis and treatment coverage), incomplete clinical and laboratory data, including viral load may limit accurate assessment of viral suppression among adolescents, potentially underestimating progress toward the third “95.” Strengthening EMR workflows, lab integration and data entry practices is therefore critical for reliable cascade monitoring.

### 4.1 Limitations

We only assessed the variables included in the assessment guide; therefore, other important clinical or lifestyle variables, such as treatment adherence, opportunistic infections beyond tuberculosis were not evaluated. Furthermore, the data may not fully capture changes over time if records were not updated or corrected to reflect shifts in clinical status. The retrospective cross-sectional design also limits our ability to establish causality or temporal trends in data quality.

The specific study context, including the EMR system in use, clinical workflows and staffing arrangements may limit the generalizability of our findings to other settings or countries. External verification of data correctness was not possible due to the paperless system. Moreover, system upgrades during the study period, such as roll out of SmartCare Pro, may have influenced data completeness and consistency across facilities.

Facilities were purposively selected to ensure variation in facility level and patient volume; however, this approach may not fully represent all health facilities in Lusaka or Zambia. No formal statistical sample size calculation was undertaken, reflecting the study’s emphasis on assessing EMR system performance rather than estimating population-level outcomes.

Finally, extracted EMR data could not be supplemented or corrected retrospectively, which may have affected measures of completeness, correctness and consistency measures. Despite these limitations, the study provides actionable insights for strengthening EMR system performance and data use in comparable low-resource public health settings.

### 4.2 Recommendations

#### 4.2.1 Implementation roadmap for strengthening EMR data quality

Based on the magnitude of observed gaps and their clinical significance, we propose the following prioritized implementation hierarchy.

##### 4.2.1.1 Priority 1: mandatory field redesign for high-gap clinical variables

Immediate system reconfiguration to enforce mandatory entry of pregnancy and breastfeeding status for all female ALHIV at each clinical encounter. Given the 96 and 88% completeness gaps respectively, this represents the highest-impact intervention.

##### 4.2.1.2 Priority 2: laboratory data integration strengthening

Strengthen automated laboratory-EMR interfaces to ensure seamless transfer of CD4 and viral load results. Where interfaces are unavailable, introduce structured prompts requiring manual entry confirmation.

##### 4.2.1.3 Priority 3: workflow optimization toward electronic-first model

Scale up real-time point-of-care entry to reduce transcription delays and improve completeness of dynamic clinical indicators.

##### 4.2.1.4 Priority 4: automated validation and logic checks

Implement system-based alerts for biologically implausible values and missing time-sensitive data (e.g., CD4 not recorded within defined intervals).

##### 4.2.1.5 Priority 5: routine data quality audits with feedback loops

Institutionalize quarterly RDQA reviews at facility level, coupled with performance dashboards to reinforce a culture of data use for decision-making.

This hierarchical approach prioritizes structural system redesign before training-only interventions, ensuring sustainable improvements in EMR data quality.

## 5 Conclusion

The EMR system for ALHIV shows high data quality in socio-demographic and treatment regimen variables, and good performance for viral load and TB history. However, substantial gaps remain in the completeness and correctness of certain clinical indicators especially CD4 counts and pregnancy/breastfeeding data. Addressing these gaps will enhance both patient management and program monitoring, and ensure that EMR data can be fully relied on for research, reporting, and decision making.

## Data Availability

The datasets presented in this article are not readily available due to in-country data protection laws, a request to share these data will need to be made to relevant authorities and once authorised, the author can share with undue reservation. Requests to access the datasets should be directed to 2930764@myuwc.ac.za.
